# The prevalence of the honeybee brood pathogens *Ascosphaera apis, Paenibacillus larvae* and *Melissococcus plutonius* in Spanish apiaries determined with a new multiplex PCR assay

**DOI:** 10.1111/1751-7915.12070

**Published:** 2013-08-06

**Authors:** Encarna Garrido-Bailón, Mariano Higes, Amparo Martínez-Salvador, Karina Antúnez, Cristina Botías, Aránzazu Meana, Lourdes Prieto, Raquel Martín-Hernández

**Affiliations:** 1 Bee Pathology Laboratory, Centro Apícola Regional (CAR)Junta de Comunidades de Castilla La Mancha, 19180, Marchamalo, Spain; 2 Epidemiology Consultant, C/Puente la Reina28050, Madrid, Spain; 3 Departamento de Microbiología, Instituto de Investigaciones Biológicas Clemente EstableMontevideo, Uruguay; 4 Animal Health Department, Facultad de Veterinaria, Universidad Complutense28040, Madrid, Spain; 5 Instituto Universitario de Investigación en Ciencias Policiales (IUICP), Comisaría General de Policía Científica, DNA LaboratoryMadrid, Spain; 6 Instituto de Recursos Humanos para la Ciencia y la Tecnología (INCRECYT), Fundación Parque Científico y Tecnológico de AlbaceteAlbacete, Spain

## Abstract

The microorganisms *Ascosphaera apis*, *Paenibacillus larvae* and *Melissococcus plutonius* are the three most important pathogens that affect honeybee brood. The aim of the present study was to evaluate the prevalence of these pathogens in honeybee colonies and to elucidate their role in the honeybee colony losses in Spain. In order to get it, a multiplex polymerase chain reaction (PCR) assay was developed to simultaneously amplify the16S ribosomal ribonucleic acid (rRNA) gene of *P. larvae* and *M. plutonius*, and the 5.8S rRNA gene of *A. apis*. The multiplex PCR assay provides a quick and specific tool that successfully detected the three infectious pathogens (*P. larvae*, *M. plutonius* and *A. apis*) in brood and adult honeybee samples without the need for microbiological culture. This technique was then used to evaluate the prevalence of these pathogens in Spanish honeybee colonies in 2006 and 2007, revealing our results a low prevalence of these pathogens in most of the geographic areas studied.

## Introduction

The brood of the honeybee (*Apis mellifera*) is susceptible to infection by a wide variety of pathogens, including *Ascosphaera apis*, *Paenibacillus larvae* and *Melissococcus plutonius*, the causative agents of some of the most important diseases affecting bees. Indeed, the fungus *A. apis* is responsible for chalkbrood disease, in which larvae are infected by ingesting fungal spores that then germinate in the digestive tract. Subsequent mycelial growth is lethal to the larvae. Dead larvae and pupae desiccated, forming mummies that contain millions of spores and that are highly infectious (Aronstein and Murray, 2010). *A. apis* is responsible for large economic losses, particularly in combination with other pathogens such as *Nosema apis* (Aydin *et al*., 2006), *Nosema ceranae* and *Varroa destructor* (Hedtke *et al*., 2011).

*P. larvae* is a gram-positive spore-forming bacterium that causes American foulbrood (AFB: Genersch *et al*., 2006). Larvae become infected by ingesting food contaminated with spores and these spores germinate in the larval midgut, the vegetative bacteria proliferating and translate to the haemocoel, killing the larvae. Dead larvae initially form a brownish, semi-fluid, glue-like colloid, and subsequently, they form highly infectious dehydrated scales (Genersch, 2010). *M. plutonius* is a gram-positive non-spore forming bacterium responsible for European foulbrood (EFB). Bacterial cells are ingested with contaminated food and reproduce within the larval midgut. Infected larvae can die before or after capping, or they may successfully pupate and form normal or undersized adults. Dead larvae are found twisted around the walls of the cell or stretched out lengthways. These larvae turn yellow, then brown and finally decompose, adopting a greyish black colour (Forsgren, 2010).

Significantly, both AFB and EFB are bacterial diseases that should be notified to the OIE (2013). These diseases have a global distribution and serious consequences, including a significant decrease in honeybee populations and honey production, which has had a strong impact on the beekeeping industry in recent years. The identification of these pathogens using classical methods involves bacteriological culture and morphological and physiological analysis. These are time-consuming processes, and the results may vary depending on the experience of the operator. However, recent advances in genetics have led to significant progress in identifying microbes, and several scientific groups have developed PCR-based techniques that can successfully identify *A. apis* (James and Skinner, 2005; Murray *et al*., 2005), *P. larvae* (Dobbelaere *et al*., 2001; Lauro *et al*., 2003) and *M. plutonius* (Djordjevic *et al*., 1998), as recommended by the OIE (2012).

Here, we describe the development of a multiplex PCR approach that significantly improves the conventional PCR techniques by incorporating multiple primers to simultaneously amplify regions of DNA from three honeybee brood pathogens in a single reaction. Given the paucity of data regarding the presence of these pathogenic agents in Spanish honeybee colonies, we used this approach to assess their distribution and prevalence in Spain in a transverse study carried out in 2006 and 2007.

## Results

### Multiplex PCR

**Figure 1:**
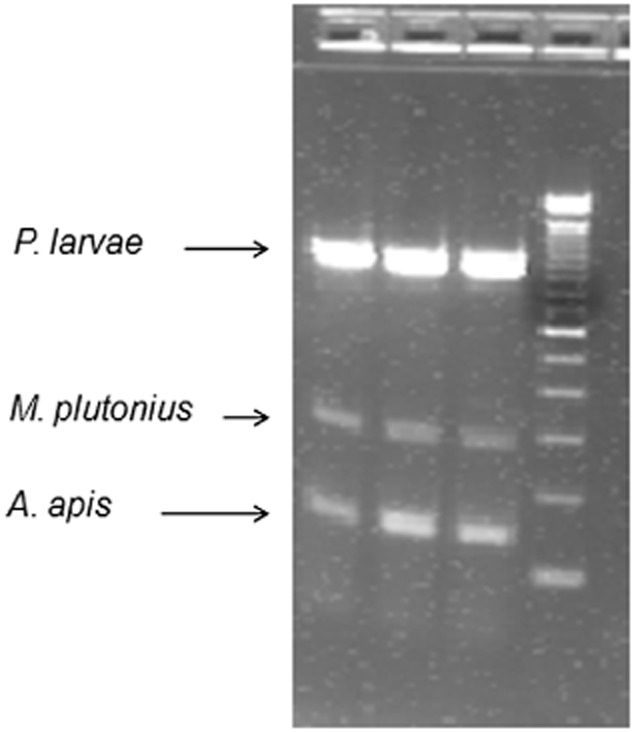
Detection of the amplicons generated by multiplex PCR in an agarose gel*: A. apis* (136 bp), *M. plutonius* (281 bp) and *P. larvae* (973 bp). Molecular weight marker 100 bp (Invitrogen). Mixed Genomic DNA from infected larvaes.

Selected primers that were designed to amplify the 5.8S ribosomal ribonucleic acid (rRNA) gene of *A. apis* and the 16S rRNA gene of *M. plutonius* and *P. larvae* (Govan *et al*., 1999) produced the expected amplicons at an annealing temperature of 59°C when DNA from reference strains for the three pathogens was analyzed, both by single and multiplex PCRs (Fig. 1). The optimum primer concentrations were defined as 0.09 μM for Ascos, 0.6 μM for Meli and 0.05 μM for *P. larvae*. The amplicons obtained for *A. apis*, *M. plutonius* and *P. larvae* exhibited a high percentage of similarity (98%, 97% and 99% respectively) with the sequences published for these pathogens. The empirical specificity was determined by multiplex PCR analysis of *Brevibacillus laterosporus* and *Paenibacillus alvei* DNA, which returned negative results.

The multiplex PCR assay described here successfully detected the presence of *A. apis*, *M. plutonius* and *P. larvae* in bee larvae exhibiting clinical symptoms of these pathogens (*n* = 2 per pathogen) (Fig. 1), while negative results were obtained with healthy larvae for all three pathogens. This assay was reproducible as when the entire process was conducted three times using pure bacterial cultures, and infected and healthy larvae, similar results were obtained each time for all the samples.

### Transverse study

The multiplex PCR was successfully used to evaluate the prevalence of *A. apis*, *M. plutonious* and *P. larvae* in Spanish apiaries. The results revealed a low prevalence of these pathogens in honeybee brood in 2006 and 2007: *A. apis* < 5%; *P. larvae* < 3%; *M. plutonius* < 1% (as reflected in Table 1). The prevalence of *A. apis* in 2007 was significantly lower than in 2006 (χ^2^ = 7.48; *P* = 0.001), although there were no differences detected in prevalence between spring and autumn in either of the 2 years analyzed (spring/autumn 2006: χ^2^ = 0.6, *P* ≥ 0.05; spring/autumn 2007: χ^2^ = 0.2, *P* ≥ 0.05).

**Table 1:** Prevalence and confidence interval (95%) of *A. apis, P. larvae* and *M. plutonius* in Spain in the transverse study

		*A. apis*		*P. larvae*		*M. plutonius*	
				
Year	Sampling	Prevalence (%)	95% CI	Prevalence (%)	95% CI	Prevalence (%)	95% CI
2006	Spring	4.8	3–6.6	1.6	0.5–2.6	0.5	0.1–1.5
	Autumn	3.7	1.5–5.8	1.5	0.5–3.6	0.3	0–1.7
2007	Spring	1.8	0.5–3.1	4.2	2.3–6	0.2	0–1.1
	Autumn	2.3	0.3–4.3	3.2	0.8–5.5	0	0–1.4

CI = confidence interval.

By contrast, the levels of *P. larvae* were significantly higher in 2007 than in 2006 (χ^2^ = 7.92, *P* < 0.05), although no differences were found between seasons (spring/autumn 2006: χ^2^ = 0.00, *P* ≥ 0.05; spring/autumn 2007: χ^2^ = 0.4, *P* ≥ 0.05).

The high sensitivity of our technique is demonstrated by its ability to detect pathogens in asymptomatic larvae. Further studies could be conducted to determine the minimum number of spores (*P. larvae* and *A. apis*) or vegetative cells (*M. plutinius*) of each agent its can be detected and determine as well the sensitivity in a more precise manner.

The detection of *M. plutonius* using the multiplex PCR represents the first reported molecular detection of this pathogen in Spain, due to no other Spanish lab had, so far, implemented this technique (MAGRAMA personal communication). However, the prevalence remained below 1% at all times analyzed, with no differences observed between years (χ^2^ = 1.34, *P* ≥ 0.05). A similar prevalence was also detected in spring and autumn (spring/autumn 2006: χ^2^ = 0.2, *P* ≥ 0.05; spring/autumn 2007: χ^2^ = 0.5, *P* ≥ 0.05).

Adult honeybee samples were analyzed in the same way as the brood samples and the prevalence of *A. apis* was similar in both types of sample (∼17%). By contrast, the detected prevalence of both *M. plutonius* and *P. larvae* was twofold greater in adult honeybees (3.5% and 71.8% respectively) than in the brood (1.2 and 33.1% respectively).

**Figure 2:**
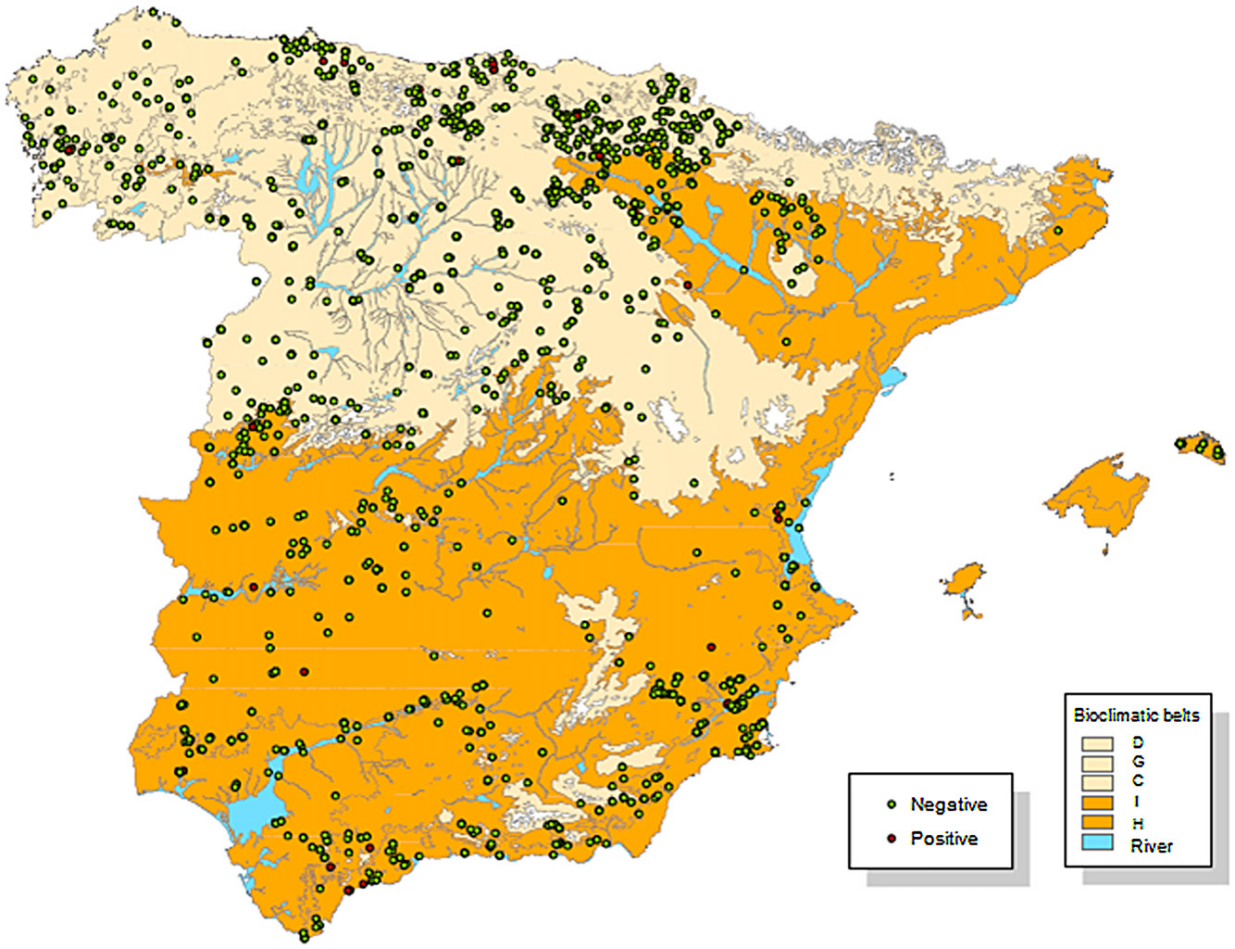
Distribution of *A. apis* in Spain according to the bioclimatic belts described by Rivas-Martínez (1987): montane (C), coline (D), supra-mediterranean (G), meso-mediterranean (H) and termo-mediterranean (I).

**Figure 3:**
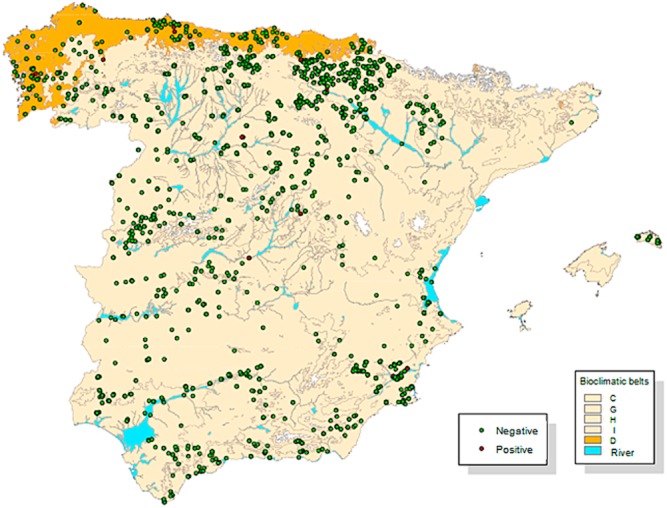
Distribution of *P. larvae* in Spain, according to the bioclimatic belts described by Rivas-Martínez (1987): montane (C), coline (D), supra-mediterranean (G), meso-mediterranean (H) and termo-mediterranean (I).

The distribution of infectious pathogens according to the climatic zones in Spain was also analyzed (see Table 2 and Fig. 2 and 3: Rivas-Martínez 1987). *A. apis* was more frequently detected in hotter areas (meso- and termo-mediterranean belts) than in warm or cold regions. By contrast, *P. larvae* was more prevalent in the coline belt, which has probable frost for 6 months per year. No significant differences were observed between the distinct climatic zones for *M. plutonius*.

**Table 2:** Distribution of *A. apis*, *P. larvae* and *M. plutonius* between the bioclimatic belts according to Rivas-Martínez (1987) classification

Bioclimatic belt	Climatic characteristic	*n*	Positive	Prevalence (%)	χ^2^	*P*	χ^2^	*P*
*Ascosphaera apis*								
Supra-mediterranean (G)	T 13 to 8°, m −1 to −4°, M 9 to 2°	263	3	1.1	–	–		
	It 210 to 60, H IX-VI Semi-arid to hyper-humid							
Montane (C)	T 12 to 6°, m 2 to −4°, M 10 to 3°	228	5	2.2	0.7	0.4129^a^		
	It 240 to 50, H IX-VI Sub-humid to hyper-humid							
Colline (D)	T > 12°, m > 2°, M > 10°	169	6	3.6	1.2	0.2804^a^		
	It > 240, H XI-IV Sub-humid to hyper-humid							
Meso-mediterranean (H)	T 17 to 13°, m 4 to −1°, M 14 to 9°	338	17	5	**4.2**	**0.0228**^a^	–	–
	It 350 to 210, H X-IV Semi-arid to hyper-humid							
Termo-mediterranean (I)	T 19 to 17°, m 10 to 4°, M 18 to 14°	173	12	6.9	**7**	**0.0081**^a^	0.69	0.6042^b^
	It 470 to350, H XII-II Arid to humid							
Missing		508						
Total		1679						
*Paenibacillus larvae*								
Supra-mediterranean (G)	T 13 to 8°, m −1 to −4°, M 9 to 2°	263	3	1.1	–	–		
	It 210 to 60, H IX-VI Semi-arid to hyper-humid							
Meso-mediterranean (H)	T 17 to 13°, m 4 to −1°, M 14 to 9°	338	4	1.2	0	0.8419^c^		
	It 350 to 210, H X-IV Semi-arid to hyper-humid							
Montane (C)	T 12 to 6°, m 2 to −4°, M 10 to 3°	228	3	1.3	0.1	0.7473^c^		
	It 240 to 50, H IX-VI Sub-humid to hyper-humid							
Termo-mediterranean (I)	T 19 to 17°, m 10 to 4°, M 18 to 14°	173	5	2.9	0.5	0.4619^c^		
	It 470 to 350, H XII-II Arid to humid							
Colline (D)	T > 12°, m > 2°, M > 10°	169	11	6.5	**5.9**	**0.0152**^c^		
	It > 240, H XI-IV Sub-humid to hyper-humid							
Missing		508						
Total		1679						
*Melissococcus plutonius*								
Montane (C)	T 12 to 6°, m 2 to −4°, M 10 to 3°	228	0	0				
	It 240 to 50, H IX-VI Sub-humid to hyper-humid							
Colline (D)	T > 12°, m > 2°, M > 10°	169	0	0				
	It > 240, H XI-IV Sub-humid to hyper-humid							
Termo-mediterranean (I)	T 19 to 17°, m 10 to 4°, M 18 to 14°	173	0	0				
	It 470 to 350, H XII-II Arid to humid							
Meso-mediterranean (H)	T 17 to 13°, m 4 to −1°, M 14 to 9°	338	1	0.3	–	–		
	It 350 to 210, H X-IV Semi-arid to hyper-humid							
Supra-mediterranean (G)	T 13 to 8°, m −1 to −4°, M 9 to 2°	263	2	0.8	0.1	0.7157		
	It 210 to 60, H IX-VI Semi-arid to hyper-humid							
Missing		488						
Total		1659						

a Prevalence of *A. apis* on each bioclimatic belt was compared with the lowest level (supra-mediterranean G).

b Prevalence of *A. apis* on termo-mediterranean (I) compared with meso-mediterranean (H)

c Prevalence of *P. larvae* on each bioclimatic belt was compared with the lowest level (supra-mediterranean G).

Bold indicates statistical significance.

T = annual average temperature, m = average of minim temperature on the colder month. M = average of maximum temperatures on the colder month. IT: Termic index = (T + m + M)x10. H: months (in Roman numbers) when frost is statistically probable to happen. Ombro climate classification according to rainfall: Arid < 200 mm, Semi-arid 200–350 mm, Dry 350–600 mm, Sub-humid 600–1000 mm, Humid 1000–1600 mm, Hyper-humid > 1600 mm.

## Discussion

Honeybees are susceptible to a wide variety of diseases and environmental threats, several of which have increased in severity in the last decade (Genersch, 2010). In the present study, we focused on pathogenic agents that affect the honeybee brood (*A. apis*, *P. larvae* and *M. plutonius*), and developed a multiplex PCR capable of detecting these pathogens in mono and co-infected colonies, even when disease symptoms are absent.

The design and optimization of multiplex PCR is more challenging than that of conventional PCR, as the annealing of multiple primers only occurs if the annealing conditions are similar for all primers and if no interference occurs between primers. The key to success for multiplex PCR is primer compatibility, careful primer design and control of the reaction conditions. We used specific primers for *A. apis* and *M. plutonius* (developed for this study) together with primers for *P. larvae* designed previously (Govan *et al*., 1999). These primers successfully amplified fragments of varying sizes (136 bp, 281 bp and 973 bp respectively), which were easily distinguished by both agarose gel and capillary electrophoresis. This technique was used successfully and reproducibly in order to analyze pure cultures of the pathogens, as well as infected larvae and adult bees.

The specificity of this technique in identifying *P. alvei* and *B. laterosporus* was also confirmed, bacterial species commonly found in apiaries that have also been associated with the development of EFB (Alippi, 1991). This approach overcomes several drawbacks associated with other molecular methods that require the isolation and pure culture of *A. apis* (James and Skinner, 2005; Murray *et al*., 2005) or *P. larvae* (Dobbelaere *et al*., 2001; Lauro *et al*., 2003), or the pre-incubation of diseased larvae in the case of EFB (Govan *et al*., 1998).

The results of our transverse study revealed a low prevalence of honeybee brood pathogens in Spain in 2006 and 2007. The prevalence of *A. apis* throughout the four sampling periods (spring and autumn of 2006 and 2007) did not exceed 5%, while that of *P. larvae* and *M. plutonius* was below 3% and 1% respectively. The prevalence of *A. apis* throughout the 2-year study was lower than that reported in other countries, such as Japan (24.1%:Yoshiyama and Kimura, 2011). Surprisingly, we observed a higher prevalence in hotter areas (meso- and termo-mediterranean belts), whose climatic characteristics are not considered conducive to the growth of this fungus. The prevalence of *Nosema ceranae* in these regions was previously reported to be significantly higher than in other areas of Spain (Martín-Hernández *et al*., 2012) and as previously suggested, it may be responsible for outbreaks of stress-related diseases such as chalkbrood (Hedtke *et al*., 2011). *A. apis* prefers conditions of high humidity combined with cool temperatures (Flores *et al*., 1996; Borum and Ulgen, 2008). Indeed, artificial warming of the hive in spring has been shown to decrease the incidence of this disease (Pederson, 1976). In addition to environmental conditions, differences in fungal strains and bee genetics may influence the incidence and severity of disease (Aronstein and Murray, 2010), and could account for its low prevalence in Spain.

*P. larvae* is considered to be a major threat to honeybees, and it is responsible for significant decreases in the colony numbers (Genersch, 2010). Although the disease caused by this bacterium progresses gradually in affected colonies, it can appear at any time of year, and it can kill infected colonies within a few months or over several years (Hansen and Brødsgaard, 1999; Genersch, 2010). Our results indicate a low prevalence of *P. larvae* in Spain during the period studied, with a significantly higher prevalence in cool regions characterized by mild winter temperatures. This result can be related with the analyzed samples in our study (samples taken randomly and asymptomatic), although they show a low prevalence of *P. larvae* spores in asymptomatic colonies in our country. While this represents the first molecular detection of *M. plutonius* in Spain, this pathogen was limited to specific apiaries. By contrast, in other European countries molecular diagnostic techniques have confirmed that EFB is endemic, such as Switzerland (Forsgren *et al*., 2005; Roetschi *et al*., 2008) and the UK (Budge *et al*., 2010).

Evaluation of the presence of *A. apis*, *P. larvae* and *M. plutonius* in adult honeybees revealed a prevalence that in the case of the bacteria was twofold higher than in brood, indicating that even in the absence of clinical signs of disease, honeybee workers can act as vector of pathogenic agents, both within the colony (Belloy *et al*., 2007) and between colonies and apiaries (Roetschi *et al*., 2008). In fact, it has been shown that worker bees are more appropriate than brood for epidemiological studies of AFB (Lindström and Fries, 2005) and EFB (Roetschi *et al*., 2008). In the case of EFB, worker bees from brood nests have bacterial loads that are approximately 20 times higher than those from the flight entrance, probably due to their contact with infected brood and their role in cleaning the cells (Roetschi *et al*., 2008).

Finally, the detection of infectious pathogens in brood samples without symptoms clearly demonstrates that they are not exclusively present in larvae that exhibit clinical signs of infection, as proposed previously (Forsgren *et al*., 2005; Belloy *et al*., 2007). The detection of these bacteria in apparently healthy brood supports the view that infected larvae can survive, pupate and emerge as adults while carrying the bacteria (Bailey and Ball, 1991). However, overall our data suggest that infectious agents targeting the brood emerge in a single event, secondary to infection by more prevalent pathogens such as *V. destructor* and *N. ceranae* (Martín-Hernández *et al*., 2012). These primary agents may be responsible for immunosuppression of the colony (Yang and Cox-Foster, 2005; Antúnez *et al*., 2009), which may then be exploited by infectious and parasitic agents, as recently described for the tracheal mite *Acarapis woodi* (Garrido-Bailón *et al*., 2012).

Therefore, the development of surveys that determine the presence of many different pathological agents in honeybee colonies can help to establish the relationship among all of them in order to correlate them with a final development of the diseases in the colonies. In the same way, these kinds of studies are basic for providing data for the establishment of sanitary policy at a country level.

## Experimental procedures

### Bacterial and fungal strains

Type strains from American Type Culture Collection (ATTC) for *A. apis* (ATCC® 38506™), *P. larvae* (ATCC® 9545™) and *M. plutonius* (ATCC® 35311™) were used as positive controls to develop the multiplex PCR assay. *B. laterosporus* (ATCC® 64™) and *P. alvei* (ATCC® 6344™), bacterial species, usually found in honeybee colonies (Djukic *et al*., 2011; 2012), were also used to confirm the specificity of the technique. Each ATCC strain was cultured individually as it is recommended. In order to activate the microorganisms, a first step in a specific liquid medium was carried out. After activation, a second step in a solid agar was performed to strain multiplication. The medium, temperature, time and atmosphere conditions for each ATCC strain are described in Table 3.

**Table 3:** Culture conditions for each reference strain

		First step			Second step			
				
Microorganism	Reference strains (ATCC)	Liquid medium	Temperature (°C)	Time	Solid medium	Temperature (°C)	Time	Atmosphere
*M. pluton*	35311	ATCC 1430 Broth	30	48–72 h	OIE (2012)	30	48–72 h	Anaerobic
*A. apis*	38506	Potato Dextrose Broth	18	48–72 h	MY-20 (Raper and Fennell, 1965)	30	7 d	Aerobic
*P. l. larvae*	9545	Brain-Heart Infusion	37	48–72 h	Blood Agar	37	48–72 h	Anaerobic
*P. alvei*	6344	Nutrient Broth	30	24–48 h	Nutrient Agar	30	24–48 h	Aerobic
*B. laterosporus*	64	Nutrient Broth	30	24 h	Nutrient Agar	30	24 h	Aerobic

### Reference brood samples

Honeybee brood with typical symptoms of chalkbrood disease (due to *A. apis* infection) and AFB (due to *P. larvae* infection) were obtained from the Centro Apícola Regional (CAR), and they were stored aseptically at −20°C. Brood samples with EFB (due to *M. plutonius*) were kindly sent to CAR by Dr. A. Roetschi. These samples served as positive controls. Asymptomatic larvae collected from healthy colonies at the CAR were used as negative controls.

### DNA extraction

Pure vegetative cell suspensions were prepared from reference bacterial strains in 1 ml of distilled water (PCR grade) for subsequent DNA extraction. Infected and healthy brood larvae were selected randomly, extracted aseptically from brood cells, and a total of 5 g was crushed for 4 min (at high velocity) in 50 ml of MilliQ H_2_O using a Stomacher machine (Stomacher 80, Seward) and plastic filter bags. Also, two samples of larvae infected with each pathogen and two samples of healthy larvae were used for subsequent DNA extraction. The macerates were centrifuged for 6 min at 800 x g, the supernatants discarded, and the pellets were re-suspended in 1 ml of distilled water (PCR grade). For DNA extraction, aliquots of each pure vegetative cell suspension and re-suspended pellets obtained from larvae (150 μl) were placed in a 96-well plate (Qiagen) containing glass beads (2 mm diameter, Sigma). At least one blank well (with water) was included for every 20 samples as a negative control of extraction. The plates were shaken in a TissueLyser machine (Qiagen), and 30 μl of ATL buffer (Cat. no. 19076, Qiagen) and 20 μl of Proteinase K (Cat. no. 19131, Qiagen) were added to each well. The plates were incubated overnight at 56°C, and DNA was subsequently extracted using a BS96 DNA Tissue extraction protocol in a BioSprint Workstation (Qiagen). The DNA obtained was frozen and stored at −20°C.

### Multiplex PCR design: methodology

Selection of target DNA: The GenBank database was searched for published DNA sequences from *P. larvae*, *M. plutonius* and *A. apis* (http://www.ncbi.nlm.nih.gov/genbank/, November, 2007). To select appropriate target loci for the PCR, the following criteria were applied: (i) sequences located in conserved regions of the genes in each species, (ii) sequences highly specific to each species were selected to avoid non-specific primer annealing and (iii) DNA fragments represented in the database by more than one individual were preferred.

For *A. apis*, 5.8S rRNA was one of the most conserved targets, and it was represented in the database by more than one individual (U68313 and U18362). At the time our study was carried out, *M. plutonius* 16S rRNA was represented by four DNA sequences (AY862507, AJ301842, X75752 and X75751) and for *P. larvae*, the primers designed by Govan and colleagues (1999) for monoplex PCR designed were used.

Primer design: DNA sequences from *A. apis* and *M. plutonius* were aligned using ClustalW to identify possible DNA polymorphisms (http://www.ebi.ac.uk/Tools/clustalw/, November, 2007) and this allowed us to avoid polymorphic points as primer binding zones. Surprisingly, alignment of the *A. apis* sequences revealed a high level of polymorphism at one end. To determine which sequence was more reliable, the sequences were aligned with the draft genome sequence for *A. apis* (Qin *et al*., 2006; http://www.hgsc.bcm.tmc.edu/projects/microbial, November, 2007), which revealed that the U68313 sequence exhibited more similarities to the draft genome sequence. This sequence was then aligned with those of other *Ascosphaera* species to select the best regions to develop a highly specific primer to detect *A. apis*: *A. duoformis* (U68316), *A. atra* (U68314), *A. xerophila* (U68326), *A. variegata* (U68319), *A. subcuticulata* (U68331), *A. solina* (U68328), *A. pollenicola* (U68329), *A. proliperda* (U68318), *A. osmophila* (U68317), *A. naganensis* (U68327), *A. major* (U68315), *A. larvas* (U68330), *A. flava* (U68332), *A. fusiformis* (U68324), *A. celerrima* (U68325), *A. colubrina* (U68320), *A. aggregata* (U68323), *A. asterophora* (U68322) and *A. acerosa* (U68321).

The *M. plutonius* sequences selected exhibited a high degree of similarity, with only punctual polymorphisms that were avoided as primer binding zones. A consensus sequence was obtained with variable sites, named using the International Union of Biochemistry code. As no other *Melissococcus* genus sequences were available in GenBank, no further alignments were performed.

In addition to their specificity, the following requirements were taken into account when choosing the primer pairs for both species: (i) that the amplicons differed in length relative to one another and to the *P. larvae* amplicon, to permit separation by agarose gel electrophoresis; (ii) that the primer sequences were suitable to amplify the DNA from the three species in a single tube (G+C content and melting temperature); and (iii) potential primer interactions are avoided (hairpin, homodimer and heterodimer structures for the six primers) to ensure the efficiency of the amplification reaction. The primers selected are shown in Table 4 and their amplicon lengths were 136 bp, 281 bp and 973 bp for *A. apis*, *M. plutonius* and *P. larvae*, respectively.

**Table 4:** Primers used in multiplex PCR for the detection of *A. apis*, *M. plutonius* and *P. larvae*

Primers	Sequence (5′-3′)	Amplicon size (pb)	Specificity
AscosFOR^a^	TGTGTCTGTGCGGCTAGGTG	136	*A. apis*
AscosREV^a^	GCTAGCCAGGGGGGAACTAA		
MeliFOR^a^	GTTAAAAGGCGCTTTCGGGT	281	*M. plutonius*
MeliREV^a^	GAGGAAAACAGTTACTCTTTCCCCTA		
Primer 1^b^	AAGTCGAGCGGACCTTGTGTTTC	973	*P. larvae*
Primer 2^b^	TCTATCTCAAAACCGGTCAGAGG		

a Primers designed in this work.

b Primers designed by Govan and colleagues (1999).

An additional specificity test was carried out by conducting a search for nearly exact matches with BLAST (http://www.ncbi.nlm.nih.gov/BLAST/, November, 2007) for each primer pair.

Single PCR was performed using the appropriate positive controls for each species and specific selected primers. A gradient PCR (58 ± 5°C) was also performed to empirically determine the annealing temperatures of the three primer pairs. The best amplicons were obtained at an annealing temperature of 59°C. Several primer concentrations were also analyzed, and the best results were obtained with concentrations of 0.09 μM for *A. apis*, 0.6 μM for *M. plutonius* and 0.05 μM for *P. larvae*. At these concentrations, we obtained the best results and avoiding primer interactions that could influence efficiency (Fig. 1)

Multiplex PCR conditions: All PCRs were carried out with a Mastercycler Ep gradient S (Eppendorf) in a 50 μl reaction mix containing: 25 μl of Fast Start PCR Master Mix (Roche Diagnostic), 0.09 μM of each Ascos primer, 0.6 μM of each Meli primer, 0.05 μM of each *P. larvae* primer, 0.4 mM of each deoxynucleoside triphosphate, 3 mMCl_2_Mg, 0.2 mg ml^−1^ bovine serum albumin, 0.1% Triton X-100 and μl of DNA template. The thermocycler program used was as follows: 95°C for 2 min; 35 cycles of 30 s at 95°C, 30 s at 59°C and 45 s at 72°C; plus a final extension step at 72°C for 7 min. Negative controls (from DNA extraction) were included in all PCR experiments, and the amplicons obtained from single and triplex PCR were visualized by electrophoresis in 2% agarose gels (E-gels; Invitrogen), and in a QIAxcel System (Qiagen) using a QIAxcel DNA High Resolution Kit (Cat. No. 929002, Qiagen) in parallel with electrophoresis size standards.

### Sequencing

*A. apis*, *M. plutonius* and *P. larvae* amplicons were purified using a QIAquick PCR purification kit (Qiagen) following the manufacturer's instructions, and they were sequenced in both directions (3730 DNA Analyzer; Applied Biosystems). The resulting sequence data was checked visually using Chromas 1.43 software, and it was then aligned and compared with the *A. apis* and *M. plutonius* consensus reference sequences, and the *P. larvae* sequence described by Govan and colleagues (1999) using ClustalW.

Multiplex PCR validation: reproducibility and specificity. To ensure that the samples used provided accurate, interpretable and reproducible results, the entire experimental process (from DNA extraction to electrophoresis) was carried out three times on different days, using pure cultures of *A. apis*, *P. larvae* and *M. plutonius* (ATCC strains), as well as infected and healthy larvae. Empirical specificity was determined by analyzing DNA extracted from other species usually found in honeybee colonies (*Brevibacillus laterosporus* and *Paenibacillus. alvei*) using our triplex PCR design.

### Transverse study: prevalence and distribution of *A. apis, P. larvae* and *M. plutonius* in honeybee larvae in Spain

The PCR method described above was used to determine the prevalence of *A. apis*, *M. plutonius* and *P. larvae* in Spanish apiaries, as part of a wider survey designed to study the phenomenon of honeybee colony loss in Spain (Garrido-Bailón *et al*., 2012; Martín-Hernández *et al*., 2012). This cross-sectional study was carried out between spring 2006 and autumn 2007, and it involved a total of 1659 asymptomatic brood samples collected from: 579 honeybee colonies in spring 2006; 319 in autumn 2006; 502 in spring 2007; and 259 in autumn 2007. In addition, a total of 84 adult bee samples (34 collected in spring 2006; 15 in autumn 2006; 29 in spring 2007; and six in autumn 2007) gathered as part of the aforementioned survey were also analyzed to determine the role of adult bees in the distribution of pathogens.

As previously described (Garrido-Bailón *et al*., 2012; Martín-Hernández *et al*., 2012), each apiary where the samples came from, were geo-referred and they were linked with the bioclimate belts described by Rivas-Martínez (1987). The distribution of the pathological agents was related to the agroclimatic information obtained and treated with Geographical Information Systems (GIS, v. 9.0, ESRI, Redlands, CA, USA). Pathogens distribution and proportions found were compared through Pearson Chi^2^ (χ^2^).

All samples were submitted to the CAR by beekeepers or veterinary services, and the brood and adult honeybee samples were stored at −20°C until they were analyzed (Garrido-Bailón *et al*., 2012; Martín-Hernández *et al*., 2012) for different pathogens.
